# Case Report: Clinical Outcome From Pallidal Stimulation in a Patient With Levodopa-Resistant Dopa-Responsive Dystonia

**DOI:** 10.3389/fneur.2022.921577

**Published:** 2022-06-06

**Authors:** Xue Wang, Shanshan Mei, Zichen Tian, Lin Wang, Guiliang Hao, Xin Zhu, Wei Mao, Jianyu Li

**Affiliations:** ^1^Department of Neurology, Xuanwu Hospital of Capital Medical University, Beijing, China; ^2^Department of Functional Neurosurgery, Xuanwu Hospital of Capital Medical University, Beijing, China; ^3^Department of Biology, Carleton College, Northfield, MN, United States; ^4^Department of Neurology, Peking Union Medical College Hospital, Chinese Academy of Medical Sciences, Beijing, China; ^5^Department of Neurology, Beijing BoRen Hospital, Beijing, China

**Keywords:** dopa-responsive dystonia, deep brain stimulation, globus pallidus internus, levodopa-resistant, GCH-I mutation

## Abstract

Dopa-responsive dystonia (DRD) is a group of movement disorders with genetic and clinical heterogeneity. Dramatic response to levodopa is the hallmark of DRD. Therefore, DRD cases with poor response to levodopa are rarely reported. In addition, the clinical outcomes from deep brain stimulation (DBS) in levodopa-resistant patients remain unclear. Here, we described the clinical outcome of pallidal stimulation in a DRD patient having a poor response to levodopa. The patient was a 25-year-old man and had a 7-year history of cervical dystonia. A novel frameshift mutation in the GCH1 gene was found in the patient as well as his elder sister and mother. Unfortunately, he had no response to a large dosage of levodopa/benserazide (600/150 mg per day) and onabotulinumtoxin A injection. Therefore, bilateral globus pallidus internus (GPi) deep brain stimulation (DBS) was performed. With parameter adjustments, the severity of his torticollis was gradually improved and relieved substantially in the 8-month follow-up visit. Our current report highlights that GPi-DBS therapy leads to promising clinical outcomes for levodopa-resistant DRD.

## Introduction

Dopa-responsive dystonia (DRD) is a group of genetically and clinically heterogeneous disorders (1). Patients with DRD harboring the GTP-cyclohydrolase 1 (GCH-1) gene mutation usually respond well to levodopa. Therefore, cases with levodopa resistant DRD were rarely reported. Few cases were reported to be treated by deep brain stimulation (DBS) (2–6). However, the clinical outcomes from DBS in levodopa-resistant patients remain unclear. Here, in this case, report, we describe the clinical outcome of pallidal stimulation in a DRD patient having a poor response to a large dosage of levodopa/benserazide (600/150 mg per day).

## Case Presentation

**Figure 1:**
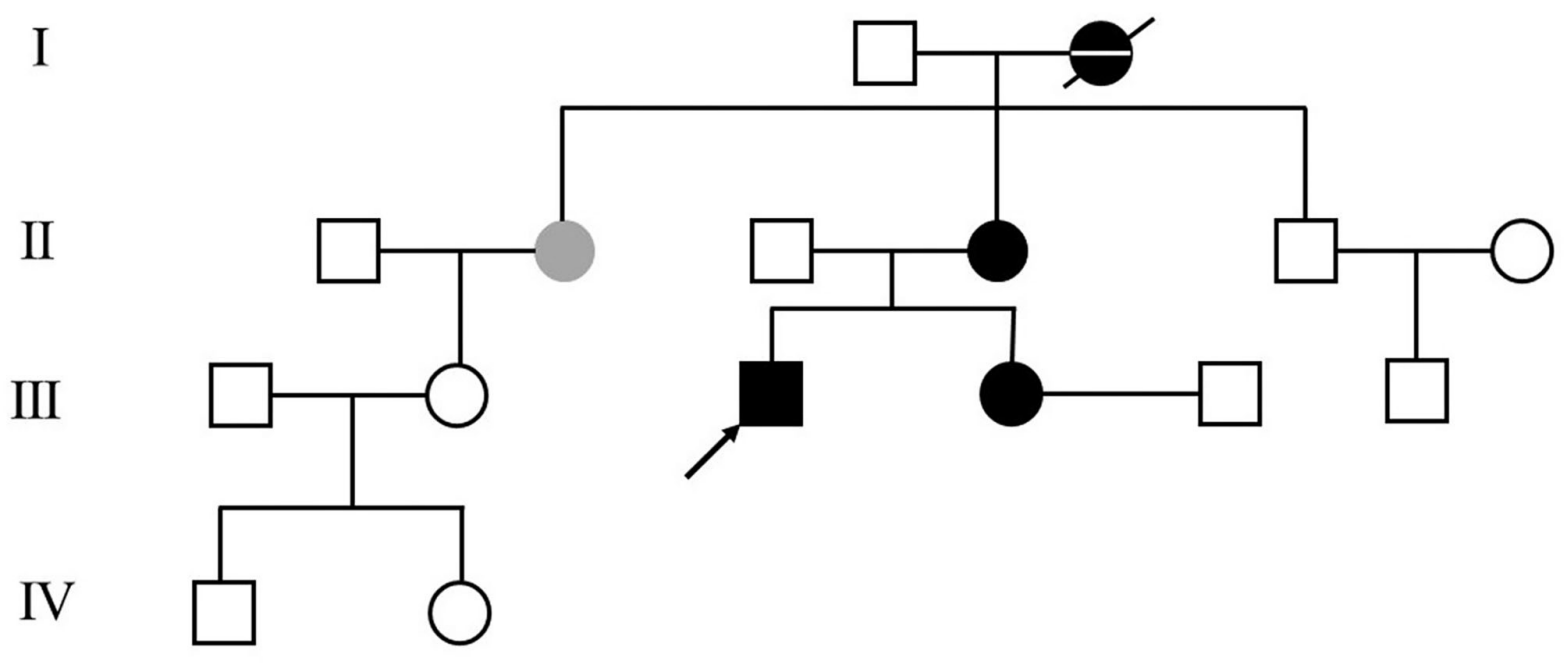
Patient's family pedigree tree. The arrow points to the presented case in this report. Black and gray color represent the family member with confirmed and with suspicious dopa-responsive dystonia (DRD) diagnoses, respectively. The family member with a confirmed diagnosis of Parkinson's disease is represented as the black dot crossed with a horizontal line.

The patient was a 25-year-old man and had a 7-year history of cervical dystonia. He presented severe left-sided torticollis (Supplementary Materials), and his symptoms can only be relieved temporarily by holding his jaw firmly, which led to severe neck pain and adversely affected his daily activities. He responded very poorly to the treatments of a large dosage of levodopa/benserazide (600/150 mg per day), baclofen, trihexyphenidyl hydrochloride, haloperidol, or on botulinum toxin A injection. In contrast to his poor response to levodopa, his 36-year-old sister, who developed abnormal postures of hands and feet at the age of 10, responded very well to 50/12.5 mg levodopa/benserazide per day. Symptoms of left-sided torticollis also occurred occasionally in his mother and his mother's only sister, but the symptoms were too mild to be treated. His grandmother was diagnosed with Parkinson's disease at age of 40 and died at age of 85. Her symptoms were upper and lower limb tremors, which were relieved by levodopa. His family pedigree tree is presented in Figure 1.

**Figure 2:**
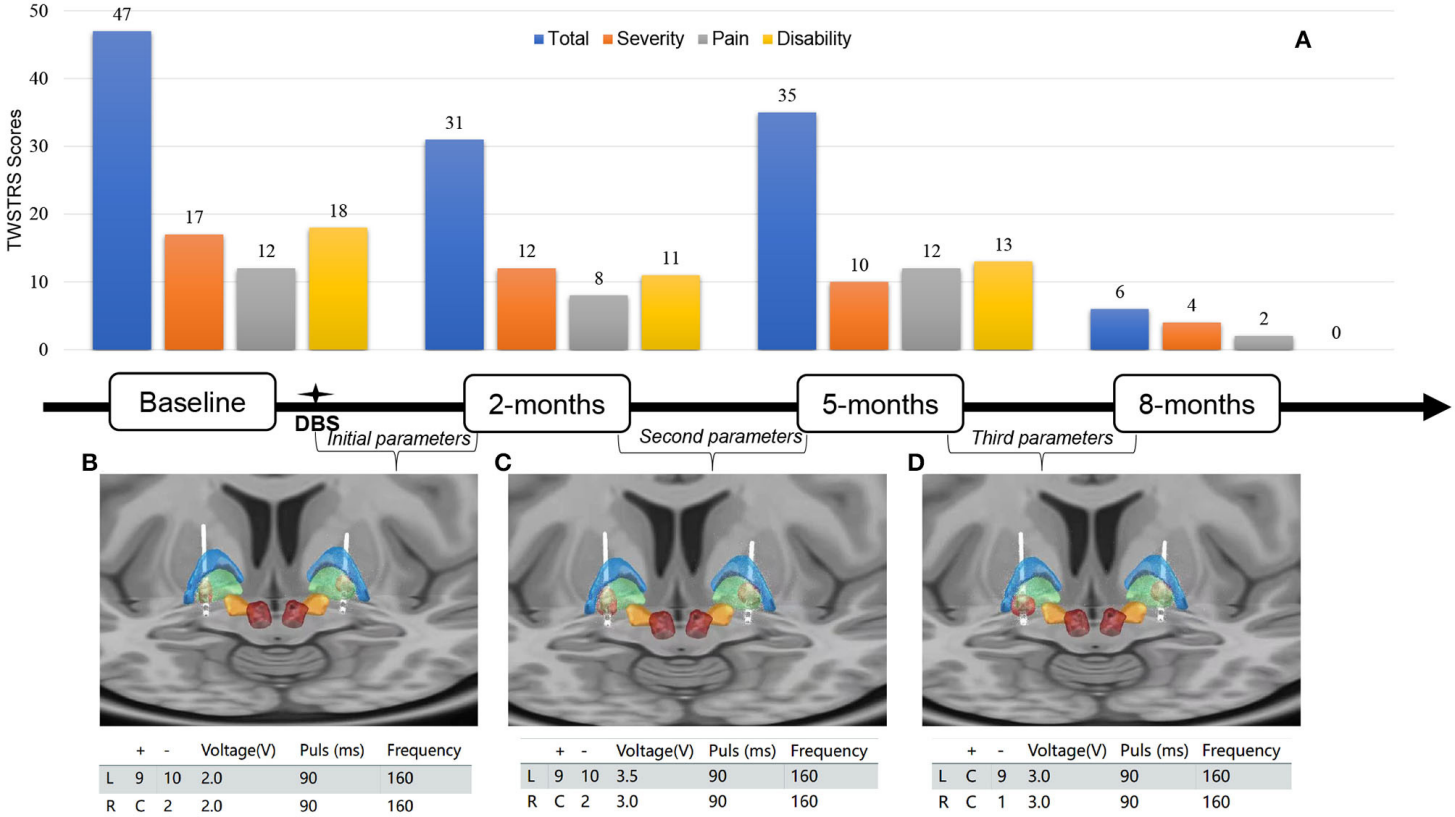
**(A)** Toronto Western Spasmodic Torticollis Rating Scale (TWSTRS) total, severity, disability, and pain scores at baseline, 2, 5, and 8 months after bilateral GPi-DBS surgery. **(B–D)** Lead location of bilateral globus pallidus internus-deep brain simulation (GPi-DBS) and volume of tissue activated (red) in the initial, second, third programming parameters. Globus pallidus internus (green), globus pallidus externus (blue), subthalamic nucleus (orange), and red nucleus (deep red). For localizing the anatomical electrode position and determining the volume of tissue activated (VTA), T1-weighted magnetization prepared rapid gradient echo (MP-RAGE) images of the patient were obtained preoperatively and registered with post-operative high-resolution CT using the Lead-DBS software package.

Neurological examination at his hospital admission revealed head torsion, neck flexion, and left shoulder elevation. His brain 18F-VMAT2 and 18F-FDG PET results were unremarkable. Whole-exome sequencing identified a frameshift mutation (c.136delA chr14-55369246 p. S46Afs^*^21) in exon 1 of the CGH-1 gene. According to the American College of Medical Genetics and Genomics (ACMG) guidance, this variant is interpreted as “pathogenic” (evidence of pathogenicity: PVS1, PS4_Supporting, and PM2). The same heterozygous mutation was also identified in his older sister and mother. The patient had a low neopterin (3.15 nmol/L, normal range is 7.24–20.41 nmol/L) level in his cerebrospinal fluid. The severity, pain, and disability of cervical dystonia were rated with Toronto Western Spasmodic Torticollis Rating Scale (TWSTRS) (Figure 2A).

Because the patient did not respond to the oral medication and botulinum toxin A injection treatments, bilateral globus pallidal internus deep brain stimulation (GPi-DBS) (3,387, Medtronic, Minneapolis, Minnesota USA) was performed. After the surgery, the patient's left-sided torticollis was slightly relieved by initiating the stimulation at voltage 2 V, pulse width 90 μs, frequency 160 Hz, and contact of left GPi (9+;10-) in bipolar mode and right GPi (C+;2-) in monopolar mode (Figure 2B). In the 2-month follow-up visit, the severity level of his torticollis was reduced by 29.4% compared with the baseline value (17 vs. 12, Figure 2A). To optimize the GPi-DBS treatment, we increased the voltage to 3.5 V on the left and 3 V on the right without changing other parameters (Figure 2C). Because the patient developed side effects, such as slurred speech and muscle twitching, in the 5-months follow-up visit, we re-adjusted the stimulation to the following setting: contact in the left GPi (C+;9-) and in the right GPi (C+;1-) in monopolar mode; the voltage at 3 V; pulse width at 90 μs; the frequency at 160 Hz (Figure 2D). Notably, during the 8-month follow-up visit, the patient's torticollis was almost in complete remission (Supplementary Materials). The global score, severity subscale, pain subscale, and disability subscale of TWSTRS were improved by 87.2, 76.5, 83.3, and 100% respectively (Figure 2D).

## Discussion

To the best of our knowledge, this is the first case report describing promising clinical outcomes from GPi-DBS treatment in a levodopa-resistant patient with DRD. As far as our literature search, only five DBS-treated DRD cases were reported. All 5 patients responded well to levodopa initially but became unresponsive gradually. The purposes of the DBS treatment in the 5 cases were to relieve the levodopa-induced motor complications (such as motor fluctuation or levodopa-induced dyskinesia) or status dystonicus. Either subthalamic nucleus deep brain stimulation (STN-DBS) or GPi-DBS was performed in these 5 cases and resulted in good postoperative outcomes, and the severity of dystonia was reduced in four cases (2–5). The severity of parkinsonism symptoms was also reduced in the case reported by Daida et al. (6) (Table 1). The randomized controlled trial has proved that GPi is an effective target for the treatment of cervical dystonia (7). Therefore, from the perspective of symptomatology, we finally chose GPi as a therapeutic target.

**Table 1:** Literature review of dopa-responsive dystonia (DRD) cases treated with deep brain stimulation (DBS).

**First author**	**Sex**	**Onset age** **(years)**	**Age at surgery (years)**	**Genotype**	**Phenotype**	**Treatment of medications**	**Purpose for surgery**	**DBS target**	**Follow-up period (months)**	**Outcome**
Dong et al. [2]	Female	5	27	TH:c. 1196C>T, PNKD: c.592C>T	Generalized dystonia **(BFMDRS: 87.5)**	Excellent effect of 300 mg levodopa/day, adding to 800mg/d by her own	motor fluctuations	B-GPi	6	**BFMDRS: 5**
Beaulieu-Boire et al. [3]	NR	7	66	NR	Generalized dystonia, Parkinsonism, **(BFMDRS-M:18; UPDRSIII-on:10)**	Levodopa, entacapone, pramipexole, selegiline, benzhexol	motor fluctuations	B-GPi	51	**BFMDRS- M(off med/on stim): 10; UPDRSIII (on/on):12**
Lobato-Polo et al. [4]	Female	7	32	GCH-1 c.671A>G	Generalized dystonia **BFMDRS-M:58**	Levodopa, clonazepam	Status dystonicus	B-STN	-	**BFMDRS-M:20**
Tormenti et al. [5]	Male	1	6	TH	Severe reflux, developmental delay, fluctuating tremor, dystonia	15mg levodopa / 12mg carbidopa every 45 minutes	LID	B-STN	14	Dystonic movements improved, speak single words clearly, dosage of levodopa reduced
Daida et al. [6]	Female	10	66	GCH-1 c.626 + 2T>G	Diurnal dystonia of the right foot. (**UPDRSIII-on:12)**	LEDD:660mg	LID and on-off phenomenon	B-STN	NR	**UPDRSIII-on:9**

*NR, Not reported; DRD, dopa-responsive dystonia; GPi, globus pallidus internus; DBS, deep brain stimulation; STN, subthalamic nucleus; TH, tyrosine hydroxylase; GCH-1, GTP cyclohydrolase I; PNKD, Paroxysmal non-kinesigenic Dyskinesia; BFMDRS, Burke-Fahn-Marsden dystonia rating scale; UPDRS, Unified Parkinson's Disease Rating Scale; LID, levodopa-induced dyskinesia*.

Patients with DRD usually have a good and sustained response to a low dosage of levodopa and only a few reports present cases showing a poor or temporary response to levodopa (8). In our current report, as suggested by Wijemanne et al. (1), a large dosage of levodopa (600 mg per day) had been used on this patient for one month, however, there was no benefit for him. A possible hypothesis for levodopa-resistance in our case could be that a prolonged dopamine deficiency in the basal ganglia disrupts the motor circuit. Genetical heterogeneity is associated with the clinical heterogeneity of DRD. Wijemanne et al. reported that patients with autosomal recessive defective GCH-1 gene required high doses of levodopa to achieve clinical effectiveness (1). In our current report, we found a novel autosomal dominant frameshift mutation in the GCH-1 gene. Tae-Beom et al. found that the prevalence of the residual signs of DRD with GCH-1 gene mutation following levodopa treatment in Korean patients was 15.8% (6/32) (9), which was similar to that of Chinese patients but higher than that of Western patients (9). The ethnic diversity of the GCH-1 gene might explain patients' various responses to levodopa.

Notably, in the current report, the patient's symptoms were relieved gradually in the first half-year after the surgery and became substantially remissive 8 months after the surgery. DBS therapy usually leads to a slow-onset improvement in motor symptoms of patients with DRD, ranging from weeks to months. Tisch et al. showed that using transcranial magnetic stimulation (TMS) paired with associative stimulation (PAS) reduced LTP-like motor cortex plasticity (10). The study by Andrea Greuel et al. demonstrated that GPi-DBS normalized dystonia-associated sensorimotor and prefrontal hyperactivity in patients with focal/segmental dystonia (11). Therefore, GPi stimulation appears to regulate distant network nodes through the basal ganglia-thalamus-cortical circuit, which may explain the slow onset of the benefits of DBS in managing dystonia.

## Conclusion

Our current report highlights that GPi-DBS therapy led to promising clinical outcomes for levodopa-resistant DRD. Proper postoperative care and long-term follow-up are essential for the successful management of DRD by DBS.

## Data Availability Statement

The original contributions presented in the study are included in the article/Supplementary Material, further inquiries can be directed to the corresponding author/s.

## Ethics Statement

Written informed consent was obtained from the individual(s) for the publication of any potentially identifiable images or data included in this article.

## Author Contributions

XW and SM were the major contributors to writing the manuscript. WM and JL contributed to the diagnosis and treatment of the patient. GH contributed to the image analysis. ZT, LW, WM, and JL contributed to checking the manuscript. All authors read and approved the final manuscript.

## Funding

The National Key R&D Program of China (No. 2020YFC2007304).

## Conflict of Interest

The authors declare that the research was conducted in the absence of any commercial or financial relationships that could be construed as a potential conflict of interest. The handling editor declared a shared parent affiliation with the authors XW, SM, GH, WM, and JL at the time of review.

## Publisher's Note

All claims expressed in this article are solely those of the authors and do not necessarily represent those of their affiliated organizations, or those of the publisher, the editors and the reviewers. Any product that may be evaluated in this article, or claim that may be made by its manufacturer, is not guaranteed or endorsed by the publisher.
